# Evidence-informed health policy 4 – Case descriptions of organizations that support the use of research evidence

**DOI:** 10.1186/1748-5908-3-56

**Published:** 2008-12-17

**Authors:** John N Lavis, Ray Moynihan, Andrew D Oxman, Elizabeth J Paulsen

**Affiliations:** 1 Centre for Health Economics and Policy Analysis, Department of Clinical Epidemiology and Biostatistics, McMaster University, 1200 Main St West, HSC-2D3, Hamilton, ON L8N 3Z5, Canada; 2 Department of Political Science, McMaster University, 1200 Main St West, HSC-2D3, Hamilton, ON L8N 3Z5, Canada; 3 School of Medicine and Public Health, Faculty of Health, the University of Newcastle, Medical Sciences Building – Level 6, Callaghan, NSW 2308, Australia; 4 Norwegian Knowledge Centre for the Health Services, Pb 7004, St Olavs plass, Oslo N-0130, Norway

## Abstract

**Background:**

Previous efforts to produce case descriptions have typically not focused on the organizations that produce research evidence and support its use. External evaluations of such organizations have typically not been analyzed as a group to identify the lessons that have emerged across multiple evaluations. Case descriptions offer the potential for capturing the views and experiences of many individuals who are familiar with an organization, including staff, advocates, and critics.

**Methods:**

We purposively sampled a subgroup of organizations from among those that participated in the second (interview) phase of the study and (once) from among other organizations with which we were familiar. We developed and pilot-tested a case description data collection protocol, and conducted site visits that included both interviews and documentary analyses. Themes were identified from among responses to semi-structured questions using a constant comparative method of analysis. We produced both a brief (one to two pages) written description and a video documentary for each case.

**Results:**

We conducted 51 interviews as part of the eight site visits. Two organizational strengths were repeatedly cited by individuals participating in the site visits: use of an evidence-based approach (which was identified as being very time-consuming) and existence of a strong relationship between researchers and policymakers (which can be challenged by conflicts of interest). Two organizational weaknesses – a lack of resources and the presence of conflicts of interest – were repeatedly cited by individuals participating in the site visits. Participants offered two main suggestions for the World Health Organization (and other international organizations and networks): 1) mobilize one or more of government support, financial resources, and the participation of both policymakers and researchers; and 2) create knowledge-related global public goods.

**Conclusion:**

The findings from our case descriptions, the first of their kind, intersect in interesting ways with the messages arising from two systematic reviews of the factors that increase the prospects for research use in policymaking. Strong relationships between researchers and policymakers bodes well given such interactions appear to increase the prospects for research use. The time-consuming nature of an evidence-based approach, on the other hand, suggests the need for more efficient production processes that are 'quick and clean enough.' Our case descriptions and accompanying video documentaries provide a rich description of organizations supporting the use of research evidence, which can be drawn upon by those establishing or leading similar organizations, particularly in low- and middle-income countries.

## Background

Learning from the experiences of existing organizations that produce clinical practice guidelines (CPGs), undertake health technology assessments (HTAs), and directly support the use of research evidence in developing health policy on an international, national, and state or provincial level (*i.e.*, government support units, or GSUs) can reduce the need to 'reinvent the wheel' and inform decisions about how best to organize support for evidence-informed health policy development processes, particularly in low- and middle-income countries (LMICs) (Table 1) [1]. We described in the second and third articles in the series the methods and findings from the survey and interview phases of our three-phase, multi-method study [2-4]. We focus here on describing the methods and findings from the study's third phase. In this phase, we produced case descriptions (based on site visits) of a purposively sampled subgroup of organizations from among those that participated in the second phase of the study and (once) from among other organizations with which we were familiar, again with an emphasis on those organizations that were particularly successful or innovative.

**Table 1:** Overview of the four-article series

[1]	Synthesis of findings from the three-phase, multi-method study
[2]	Survey of a senior staff member (the director or his or her nominee) of clinical practice guideline-producing organizations, HTA agencies, and government support units

[3]	Interview with the senior staff member of a purposively sampled subgroup of these three types of organizations, with an emphasis on those organizations that were particularly successful or innovative

**This article**	**Case descriptions (based on site visits) of one or more organizations supporting the use of research evidence from among the cases described in the interviews and (once) other cases with which we were familiar, again with an emphasis on those organizations that were particularly successful or innovative**

Previous efforts to produce case descriptions in this field have focused on topics like: 1) the use of research evidence in particular policy decisions, rather than the GSUs that may have produced the research evidence and supported its use [5-11]; and 2) the research evidence on specific technologies [12-14], or HTAs in specific jurisdictions [15,16], rather than on the HTA agencies that may have produced the research evidence or HTAs and supported their use. Moreover, although numerous CPG-producing organizations and HTA agencies have had external evaluations [17-19], these evaluations have typically been reported in unpublished internal documents and they have not used a common approach or been analyzed as a group to identify the lessons that have emerged across multiple evaluations.

Case descriptions offer the potential for capturing the views and experiences of many individuals who are familiar with an organization, including staff, advocates, and critics. Moreover, case descriptions offer the potential to focus on organizations that are of significant interest yet have been understudied, namely GSUs, organizations that are in some way successful or innovative, and organizations that are based in LMICs. We decided during the course of the study to make short video documentaries about each case, and a cameraperson/editor/technical producer was hired to work with a member of the study team (RM) on this series. Video documentaries offer the potential for 'bringing alive' the case descriptions in ways that text rarely can.

## Methods

### Study sample

We purposively sampled a subgroup of organizations from among those that participated in the second (interview) phase of the study and (once) from among other organizations with which we were familiar, again with an emphasis on those organizations that were particularly successful or innovative. We used the same three criteria used in the second phase of the study and added four additional criteria: 1) coverage of both low- and middle-income countries, with a particular emphasis on low-income countries; 2) coverage of all major regions, with a particular emphasis on Africa, Asia, and Latin America; 3) coverage of the three categories of organizations, with a particular emphasis on GSUs; and 4) coverage of the themes that emerged from the survey and interviews. One organization was selected based on our knowledge of the field, rather than the survey or interviews – the Regional East African Community Health (REACH) Policy Initiative, which is currently in the resource-mobilization phase of its development. One member of the study team (RM) applied the first criterion (*i.e.*, able to provide rich descriptions of lessons learned) and three members of the study team (AO, JNL, RM) applied the remaining criteria, first independently and then as a group.

### Case description data collection protocol development and site visits

We developed the first draft of the case description data collection protocol after having conducted preliminary analyses of both the questionnaires and interviews. The protocol included the types of individuals with whom interviews were to be requested, the interview guide, and the sorts of images to be captured in the video documentaries. The types of individuals with whom interviews were requested included one to two staff members other than the director of the organization, an advocate of the organization, and at least one critic of the organization. Sometimes the individuals we interviewed were based in other organizations and even in other countries, so the case descriptions vary in whether they focus on a single organization or on a set of interlinked organizations with our sampled organizations as our main focus. Publicly available documents pertinent to the site visits were also requested and gathered.

The interview guide included four core questions – strengths, weaknesses, advice for others, and suggestions for the World Health Organization (WHO) – that were followed by organization-specific questions that arose based on responses provided in the questionnaire and interviews and by cross-cutting questions that addressed particular themes or hypotheses that emerged from the survey or interviews. We piloted the interview guide with one organization chosen for a site visit. No significant changes were made after piloting. One member of the study team (RM) and the cameraperson conducted all the site visits. A request to host a site visit was sent by email to the director of each selected organization (or other staff) and the arrangements were made through e-mail or telephone calls. Most interviews were videotaped, but only select interview segments were transcribed verbatim. For a small number of interviews with people in the field, only notes were taken. The list of images to be captured included city panoramas, the buildings in which the organization is located, the reception desk, key interviewees, and other images to help illustrate the narrative of each case description.

### Data management and analysis

Detailed summaries of each case description were prepared by one member of the study team (RM) using the videotapes, notes taken during the interviews, notes taken during the visit, and documents obtained during the visit, and these detailed summaries were subsequently analyzed independently by two members of the study team (AO, JL). The detailed summaries were organized by question and any additional points raised during the visits were grouped together at the end of each summary. Themes were identified in both the full interviews and the answers to the four key questions, using a constant comparative method of analysis. Then question- and theme-specific groupings of the detailed summaries were read and the themes modified or amplified. Illustrative quotations were identified to supplement the narrative descriptions. We then produced a brief (one to two page) description for each case. One member of the study team (RM) and the cameraperson/editor produced and edited short video documentaries to accompany each case description.

The principal investigator for the overall project (AO), who is based in Norway, confirmed that, in accordance with the country's act on ethics and integrity in research, this study did not require ethics approval from one of the country's four Regional Committees for Medical and Health Research Ethics. We obtained verbal consent to participate in an interview and to have the interview videotaped for possible later incorporation in a video documentary. The nature of our request to participate in an interview, and our site visit more generally, made clear that we would be profiling particular organizations. The nature of our request to participate in an interview, and videotaping of the interview more generally, made clear that participants' comments could be attributed directly to them. We did not in any way indicate that we would treat interview data as confidential or that we would safeguard participants' anonymity. We shared a report on our findings and the video documentaries with participants and none of them requested any changes to how we present the data or to the video-recordings.

## Results

The director and one to two staff members, an advocate, and at least one critic were interviewed as part of each of the eight site visits, for a total of 51 interviews. A majority of the organizations were GSUs and based in Africa (two directly and one indirectly through a North-South partnership), Asia (two) or Latin America (two) (Table 2 – see Additional files 1, 2, 3, 4, 5, 6, 7, 8, and 9). Only one individual declined to participate in the interviews conducted as part of the site visits. Organizations and their advocates and critics highlighted a number of key strengths and weaknesses of the organizations selected for more detailed study, provided advice that could be offered to other organizations trying to support the use of research evidence in developing CPGs, HTAs, and health policy, and made suggestions for WHO (and for other international organizations and networks) about how it can facilitate this work. The case descriptions are remarkably varied in the themes that they explore. We highlight here the themes that emerged in two or more cases. (Both the case descriptions and video documentaries are available for viewing on the journal website.)

**Table 2:** Case descriptions and the length of the video documentaries

Case	Brief description	Length (minutes: seconds)	Weblinks
	A short introduction to the eight case descriptions	1:30	AF 4-1

REACH Policy Initiative, East Africa	An initiative to create a multi-national unit that will act as a bridge between research and policy in the East African Community (comprising Kenya, Tanzania, and Uganda)	8:26	AF 4-2

Thailand	A constellation of research units that informed the development and evaluated the implementation of Thailand's nascent universal health insurance program, known popularly as the 30 Baht scheme	7:46	AF 4-3

Free State, South Africa	A set of long term relationships between provincial policy-makers and researchers and the tensions that can arise in these relationships	9:55	AF 4-4

Pharmaceutical Benefits Scheme, Australia and South Africa	An evidence-based drug assessment and pricing scheme in Australia and South Africa	9:18	AF 4–5

Philippines	An initiative to address conflicts of interest and inequity in the production of clinical practice guidelines	9:01	AF 4–6

Chile	An initiative to use clinical practice guidelines to make the best use of scarce resources	7:48	AF 4–7

National Institute for Health and Clinical Excellence (NICE), United Kingdom	A unit producing guidelines and health technology assessments with a new focus on producing evidence-based pubic health guidelines to address health inequalities	6:12	AF 4–8

Mexico	A comprehensive effort to draw on research evidence to inform the development, implementation and evaluation of the new health insurance scheme	8:41	AF 4–9

Two organizational strengths were repeatedly cited by individuals participating in the site visits – use of an evidence-based approach, and existence of a strong relationship between researchers and policymakers – although each strength brought with it a related challenge (the time-consuming nature of an evidence-based approach, and the need to manage the conflicts of interest that can emerge in any close relationship between researchers and policymakers). The examples of using an evidence-based approach are quite diverse: 1) employing an evidence-based approach to drug assessment and prescribing (in Australia and South Africa); 2) adopting an evidence-based CPG development process that addresses equity as well as effectiveness and efficiency (in the Philippines); 3) relying on systematic reviews of the research literature as a way to protect against vested interests influencing the identification, selection, appraisal, and synthesis of research evidence (in Chile); 4) using tried and tested methods that are appropriate to the questions asked (in the United Kingdom); and 5) drawing on health systems research to inform debate and legislation and incorporating prospective evaluations as part of national health reform (in Mexico). The strong relationship between researchers and policymakers came in the form of both traditional relationships (in Mexico, the Philippines, South Africa, and Thailand) and in the form of some researchers becoming policymakers themselves, which allowed them to bring to the policymaking process their knowledge of research evidence and their contacts within the research community (in Mexico, the Philippines, and Thailand). Site visit participants from east Africa offered several unique perspectives on these relationships: 1) a home-grown model will have a greater likelihood of success; 2) high-level political support is needed for any mechanism that purports to help decision-makers make more informed decisions about health systems; and 3) an intermediary that can broker relationships between researchers and policymakers constitutes a promising mechanism.

Other strengths were cited less frequently. Site visit participants from only three organizations explicitly identified as a strength their organizations' efforts to produce highly relevant products (such as operational research, systematic reviews, CPGs, or HTAs), proactively disseminate these products, or facilitate access to them. In South Africa, their focus on operational research to guide program development was cited as a strength. In Thailand, their focus on both operational research and proactively disseminating this research was cited as a strength. And in east Africa their focus on operational research and systematic reviews, as well as their efforts to proactively disseminate this research evidence, and facilitate access to it, was cited as a strength. Similarly, site visit participants from only three organizations explicitly identified capacity, and specifically long-term investments in human and/or institutional resources, as a strength. Participants from the Philippines focused on human resources, whereas participants from Mexico and Thailand focused on both human and institutional resources. Participants from two organizations singled out independence or impartiality as a strength: the Philippines in CPG development processes, and Thailand in research generally but also specifically in policy evaluation where they considered independence and impartiality as protections against bias. Participants from two organizations focused on North-South partnerships as a strength, with such partnerships well-established in Australia (for example, with Iran and South Africa) and with North-North partnerships established and North-South partnerships only now emerging in the United Kingdom.

Two organizational weaknesses – a lack of resources and the presence of conflicts of interest – were repeatedly cited by individuals participating in the site visits. The lack of both financial and human resources was seen as a weakness in east Africa, South Africa and Thailand, with east African participants in the site visit highlighting that the lack of resources gave donors an influential role in setting the organization's direction, and with South African participants highlighting the lack of time that can be given by key human resources. Participants from the Philippines emphasized a lack of financial resources, whereas Chilean participants emphasized a lack of human resources. Conflicts of interest were seen as a major and critical issue in six of the eight countries, however, the context in which these conflicts emerge or how they are expressed varies significantly across countries. Thai participants pointed out that having researchers in very close relationships with policymakers can lead to distortions in their research, and that having researchers housed within institutions wholly funded by the Ministry of Health can raise concerns if their independent research contradicts or challenges policymakers. South African participants noted that tension has arisen between researchers and policymakers in their country. Australian participants cited attacks by the pharmaceutical industry, and participants from the Philippines pointed out that pharmaceutical company actions and medical equipment ownership can affect clinicians' behaviours. Participants from the United Kingdom indicated that stakeholders can learn how to 'get around' processes, and one Mexican participant indicated that politicians can select comparisons that make them or their jurisdiction look good. However, it is important to point out that many of these conflicts of interest are almost always hypothetical, and in only one case – the Philippines – are there ongoing challenges in managing it.

Other weaknesses were cited less frequently. Participants from two organizations explicitly identified as a weakness their efforts to proactively disseminate their products (United Kingdom), facilitate access to them or both (Mexico). Also, participants from many organizations cited sector-specific weaknesses. For example, participants in a site visit of an Australian organization focused on the pharmaceutical sector identified: 1) the need to look at affordability, not just cost-effectiveness, in developing countries; 2) the need to look at classes of drugs, not each drug individually, to be more efficient; 3) the reality that new drugs have to be compared to old drugs; and 4) the reality that policymakers sometimes find out later that a drug had advantages or disadvantages that weren't apparent at time of assessment.

Site visit participants frequently offered two types of advice to those establishing or working in other similar organizations: 1) learn from other organizations (which was supported by participants from Australia, east Africa, Mexico, South Africa, and the United Kingdom); and 2) develop capacity among and retain skilled staff and collaborators (which was supported by participants from Australia, Chile, Mexico, Philippines, and Thailand). While participants from only two organizations (those located in South Africa and Thailand) explicitly recommended that others focus on getting researchers and policymakers to work together, this advice was implicit in the comments of participants from all organizations. Other advice included: 1) involving the full array of stakeholders in any discussions about setting up new organizations or new mechanisms within existing organizations (recommended by participants from east Africa and the United Kingdom); 2) getting the processes or methods right from the beginning (recommended by participants from Mexico and the United Kingdom); 3) obtaining strong political commitment (recommended by participants from Australia although this advice was implicit in the comments made by almost all organizations); and 4) considering equity (recommended by participants from the Philippines although this point was made implicitly by participants from the United Kingdom).

Participants offered a number of suggestions for WHO (and for other international organizations and networks), however, only two suggestions were offered with any frequency. Participants from five organizations suggested that WHO play a role in mobilizing one or more of government support, financial resources, and the participation of both policymakers and researchers. Participants from east Africa and Thailand spoke to all three of these roles whereas participants from Australia emphasized mobilizing government support and financial resources, participants from Mexico emphasized mobilizing government support and the support of WHO representatives, and participants from both South Africa and the United Kingdom emphasized mobilizing government support. Participants from three organizations suggested that WHO play a role in creating knowledge-related global public goods. Participants from Mexico emphasized WHO's role in developing and promoting conceptual frameworks, standardized methods, and comparative analyses. Participants from the United Kingdom, on the other hand, recommended that WHO set up the evidence synthesis component of their country's National Institute for Clinical Excellence for LMICs to use as an input into their own CPG and HTA production processes. Participants from WHO made a somewhat similar point (albeit more implicitly), but they placed the emphasis more on WHO facilitating country collaborations to achieve the same goal. The other advice offered to WHO (and other international organizations and networks) included: 1) avoid developing global CPGs (the Philippines); 2) lend credibility and support to national CPG development processes (the Philippines); 3) create awareness about the need for free online access to journals in middle-income (as well as low-income) countries (Chile); 4) provide training in use of evidence-based methods (Chile); and 5) issue a general call to develop a more sophisticated understanding of causation and of social inequality (United Kingdom).

## Discussion

### Principal findings from the case descriptions

Two organizational strengths were repeatedly cited by individuals participating in the site visits – use of an evidence-based approach and existence of a strong relationship between researchers and policymakers – although each strength brought with it a related challenge (the time-consuming nature of an evidence-based approach and the need to manage the conflicts of interest that can emerge in any close relationship between researchers and policymakers). Two organizational weaknesses – a lack of resources and the presence of conflicts of interest – were repeatedly cited by individuals participating in the site visits. Site visit participants frequently offered two types of advice to those establishing or working in other similar organizations: learn from other organizations, and develop capacity among and retain skilled staff and collaborators. While participants from only two organizations explicitly recommended that other organizations focus on getting researchers and policymakers to work together, this advice was implicit in the comments of participants from all organizations. Participants offered a number of suggestions for WHO (and for other international organizations and networks), however, only two suggestions were offered with any frequency. Participants from five organizations suggested that WHO play a role in mobilizing one or more of government support, financial resources, and the participation of both policymakers and researchers. Participants from three organizations suggested that WHO play a role in creating knowledge-related global public goods.

### Strengths and weaknesses of the case descriptions

The case descriptions have four main strengths: 1) a majority of the organizations were GSUs and based in Africa, Asia or Latin America; 2) we drew on a regionally diverse project reference group to ensure that our case description data collection protocol was fit for purpose; 3) we drew on 51 interviews, documentary analyses, and previously collected data (from phases one and two) to produce the case descriptions; and 4) only one individual declined to participate in the interviews conducted as part of the site visits. The case descriptions have one main weakness, which they share with the other two phases in the study: despite efforts to ask questions in neutral ways, many organizations may have been motivated by a desire to tell us what they thought we wanted to hear (*i.e.*, there may be a social desirability bias in their responses).

### What the case descriptions add

The findings from our case descriptions, the first of their kind, intersect in interesting ways with the messages arising from two systematic reviews of the factors that increase the prospects for research use in policymaking [20,21]. First, one finding – that the existence of a strong relationship between researchers and policymakers emerged as one of two frequently identified organizational strengths – bodes well given both systematic reviews concluded that interactions between researchers and policymakers increase the prospects for research use. On the downside, the corresponding challenge of needing to manage the conflicts of interest that can emerge in any close relationship between researchers and policymakers suggests that more attention needs to be given to this domain [22]. Second, another finding – that an evidence-based approach was the second of two frequently identified organizational strengths, but that the time-consuming nature of this approach was seen as a closely related challenge – bodes less well given the more recent of the two systematic reviews concluded that timing and timeliness increase the prospects for research use [20,21]. This suggests that more attention needs to be given to developing more efficient production processes that are 'quick and clean enough' (as opposed to 'quick and dirty') [23].

The advice being offered to WHO (and to other international organizations and networks) – mobilizing one or more of government support, financial resources, and the participation of both policymakers and researchers, as well as creating knowledge-related global public goods – appears highly germane. WHO has mobilized government support and the participation of both policymakers and researchers through the Evidence-Informed Policy Networks that it has sponsored [24], however, it has yet to mobilize the financial resources to sustain them. And WHO has recently begun to take important steps to address the deficiencies that were identified in its production of knowledge-related global public goods [25].

### Implications for policymakers and for international organizations and networks

Policymakers have a central role to play in helping organizations balance the need for strong relationships between researchers and policymakers and the need for independence and managing conflicts of interest. Moreover, if policymakers wish to be able to draw on high quality research evidence to inform policymaking processes, they will need to provide the resources necessary to sustain these organizations. WHO and other international organizations and networks have a key advocacy role to play in helping to mobilize one or more of government support, financial resources, and the participation of both policymakers and researchers. These organizations and networks also have a key leadership role to play in enhancing their capacity to create knowledge-related global public goods.

### Implications for future research

As we argued in the second article in the series, there is a need for establishing a common framework for evaluations of the impact of these organizations, not just providing case descriptions as we have done, in order to further promote cross-organization and cross-jurisdiction learning. And as we argued in the third article in the series, there is also a need for research about methods and organizational structures to respond rapidly to policymakers' questions, and for research about balancing the need for strong links with policymakers on the one hand and the need for independence and managing conflicts of interest on the other.

## Competing interests

The authors declare that they have no financial competing interests. The study reported herein, which is the third phase of a larger three-phase study, is in turn part of a broader suite of projects undertaken to support the work of the World Health Organization (WHO) Advisory Committee on Health Research (ACHR). Both JL and AO are members of the ACHR. JL is also President of the ACHR for the Pan American Health Organization (WHO's regional office for the Americas). The Chair of the WHO ACHR, a member of the PAHO ACHR, and several WHO staff members were members of the project reference group and, as such, played an advisory role in study design. Two of these individuals provided feedback on the penultimate draft of the report on which the article is based. The authors had complete independence, however, in all final decisions about study design, in data collection, analysis and interpretation, in writing and revising the article, and in the decision to submit the manuscript for publication.

## Authors' contributions

JL participated in the design of the study, participated in analyzing the qualitative data, and drafted the article and the report on which it is based. AO conceived of the study, led its design and coordination, participated in analyzing the qualitative data, and contributed to drafting the article. RM participated in the design of the study, led the data collection and the analysis of the qualitative data, and contributed to drafting the article. EP contributed to data collection. All authors read and approved the final manuscript.

## Supplementary Material

Additional file 1 **Introduction.** A short video introduction to the eight case descriptions. Click here for file

Additional file 2 **REACH Policy Initiative, East Africa.** A video documentary about an initiative to create a multi-national unit that will act as a bridge between research and policy in the East African Community (comprising Kenya, Tanzania, and Uganda) Click here for file

Additional file 3 **Thailand.** A video documentary about a constellation of research units that informed the development and evaluated the implementation of Thailand's nascent universal health insurance program, known popularly as the 30 Baht scheme. Click here for file

Additional file 4 **Free State, South Africa.** A video documentary about a set of long term relationships between provincial policy-makers and researchers and the tensions that can arise in these relationships. Click here for file

Additional file 5 **Pharmaceutical Benefits Scheme – Australia and South Africa.** A video documentary about an evidence-based drug assessment and pricing scheme in Australia and South Africa. Click here for file

Additional file 6 **Philippines.** A video documentary about an initiative to address conflicts of interest and inequity in the production of clinical practice guidelines. Click here for file

Additional file 7 **Chile.** A video documentary about an initiative to use clinical practice guidelines to make the best use of scarce resources. Click here for file

Additional file 8 **National Institute for Health and Clinical Excellence (NICE), United Kingdom.** A video documentary about a unit producing guidelines and health technology assessments with a new focus on producing evidence-based pubic health guidelines to address health inequalities. Click here for file

Additional file 9 **Mexico.** A video documentary about a comprehensive effort to draw on research evidence to inform the development, implementation and evaluation of the new health insurance scheme. Click here for file
